# Integrated analysis of a competing endogenous RNA network in renal cell carcinoma using bioinformatics tools

**DOI:** 10.1042/BSR20190996

**Published:** 2019-07-05

**Authors:** Wei-dong Jiang, Zhi-hua Ye

**Affiliations:** Department of Urology and Hubei Key Laboratory of Kidney Disease Pathogenesis and Intervention, Huangshi Central Hospital, Affiliated Hospital of Hubei Polytechnic University, Edong Healthcare Group, Huangshi, Hubei 435000, P.R. China

**Keywords:** bioinformatics analysis, circRNA, competitive endogenous RNA, microRNA, Renal cell carcinoma

## Abstract

Background: Circular RNAs (circRNAs) are known to be closely involved in tumorigenesis and cancer progression. Nevertheless, their function and underlying mechanisms in renal cell carcinoma (RCC) remain largely unknown. The aim of the present study was to explore their expression, functions, and molecular mechanisms in RCC.

Methods: We downloaded the circRNA expression profiles from Gene Expression Omnibus (GEO) database, and RNA expression profiles from The Cancer Genome Atlas (TCGA) database. A ceRNA network was constructed based on circRNA–miRNA pairs and miRNA–mRNA pairs. Interactions between proteins were analyzed using the STRING database, and hub genes were identified using the cytoHubba app. We also constructed a circRNA–miRNA–hub gene regulatory module. Functional and pathway enrichment analyses were conducted using “DAVID 6.8” and R package “clusterProfiler”.

Results: About 6 DEcircRNAs, 17 DEmiRNAs, and 134 DEmRNAs were selected for the construction of ceRNA network of RCC. Protein–protein interaction network and module analysis identified 8 hub genes. A circRNA–miRNA–hub gene sub-network was constructed based on 3 DEcircRNAs, 4 DEmiRNAs, and 8 DEmRNAs. GO and KEGG pathway analysis indicated the possible association of DEmRNAs with RCC onset and progression.

Conclusions: These findings together provide a deeper understanding of the pathogenesis of RCC and suggest potential therapeutic targets.

## Introduction

Renal cell carcinoma (RCC) is one of the most common urological malignancies, accounting for 2–3% of all adult tumors, and is only secondary to bladder cancer. Its incidence is steadily increasing every year [1,2]. Despite the recent efforts in multimodal approaches, the prognosis of patients with RCC remains poor. This situation is mainly due to delayed diagnosis, high-frequent metastasis, and recurrence after surgery [3,4]. Therefore, it is important to further explore the mechanism of RCC and its metastasis, to obtain new insights in improving relevant therapy and exploring new therapeutic targets.

Circular RNAs (circRNAs) are formed by covalent closure of the 3′ and 5′ ends of an RNA molecule. Since circRNAs do not have 5′ or 3′ ends, they are resistant to exonuclease-mediated degradation and are presumed to be stabler than most linear RNAs in cells [5–8]. CircRNA is abundant in eukaryotic cells, highly conserved, and structurally stable, with a certain degree of organization, timing, and disease-specificity [7,8]. Due to these features, circRNAs have become new hotspots for research. Numerous studies have shown circular RNAs to play important regulatory roles in the development of tumor [9–11]. circRNAs have been reported to contain multiple miRNA-binding sites, with which they bind to miRNAs, also defined as ‘miRNA sponges’, leading to the inhibition of activity of miRNAs and regulation of expression of their downstream target genes [12–14]. Liu et al. [12] found circRNA hsa_circ_0008039 to be highly expressed in breast cancer (BC) tissues. Circ-VANGL1 silencing inhibited cell proliferation, arrested cell-cycle progression, and reduced migration both *in vitro* and *in vivo*. E2F3 is a downstream mRNA target of miR-432-5p, and its expression is positively regulated by hsa_circ_0008039 and negatively by miR-432-5p expression in BC cells. Therefore, hsa_circ_0008039/miR-432-5p/E2F3 axis was considered as a novel essential signaling pathway involved in BC progression. Furthermore, CEP128 acts as a ceRNA to regulate SOX11 by sponging miR-145-5p, thereby reducing the inhibitory effect of miR-145-5p on SOX11 in bladder cancer [13].

In our study, we performed a joint analysis considering the array-based and sequence-based data of RCC. We successfully constructed the circRNA–miRNA–mRNA and circRNA–miRNA–hub gene networks. Furthermore, we also performed a series of analyses including protein–protein interaction (PPI) analysis and functional enrichment analysis.

## Materials and methods

### Data collection

We screened the expression profiles of circRNA in GEO datasets (http://www.ncbi.nlm.nih.gov/gds/) available till January 2019. The following strategy was used: (circular RNA or circRNA) and (renal cell cancer or renal cell carcinoma). We selected data according to the following criteria: each dataset included RCC tissue and adjacent normal tissues, and each group contained more than 3 samples. The GSE100186 dataset included 4 normal renal tissues and 4 RCC tissues. We also downloaded RCC transcriptome profiles from the TCGA database. Besides, the miRNAseq and mRNAseq data were also downloaded using the Data Transfer Tool (provided by GDC Apps) (https://tcga-data.nci.nih.gov/). The miRNA profiles contained 905 RCC tissues and 130 adjacent normal renal tissues, and the mRNA profiles contained 895 RCC tissues and 128 adjacent normal tissues. The R software package was used to process the downloaded files and to convert and reject the unqualified data. The data were calibrated, standardized, and log2 transformed. No ethical approval or informed consent was required in the present study since we used the publicly available data from GEO and TCGA.

### Differential expression analysis

The differently expressed circRNAs (DEcircRNAs) were screened using Limma package, with the criterion of |log 2 (fold change [FC])| > 2 and adjusted *P*-value < 0.01. Additionally, the edgeR package was used to screen differentially expressed miRNA (DEmiRNA) and mRNA (DEmRNA) with thresholds of |log 2 (fold change [FC])| > 1 and adjusted *P*-value < 0.05.

### Construction of the ceRNA network

We used the Circular RNA Interactome (CircInteractome) (https://circinteractome.nia.nih.gov/) database to predict the miRNA-binding sites (MREs). The target miRNAs were compared to DEmiRNA based on The Cancer Genome Atlas (TCGA); only overlapping genes were selected as candidate genes. Next, we used miRTarBase and TargetScan databases [15,16] to predict interactions between miRNA and mRNA. Only the mRNAs recognized by both databases were considered as candidate mRNAs, and were intersected with DEmRNAs to screen the DEmRNAs targeted by DEmiRNAs. The circRNA–miRNA–mRNA regulatory network was constructed using a combination of circRNA–miRNA pairs and miRNA–mRNA pairs. Finally, the network was visualized and mapped using Cytoscape v3.7.0.

### Construction of PPI network and module analysis

To assess the interactions between DEmRNAs, we constructed a PPI network using the Search Tool for the Retrieval of Interacting Genes (STRING) online tool, which can provide comprehensive interactions among proteins and genes. The cut-off criteria included a combined score of > 0.9 for a PPI network and a node degree of ≥ 3 for screening hub genes. We next used the cytoHubba app to select modules of hub genes from the PPI network. The interaction network was visualized using Cytoscape software.

### Gene Ontology and pathway enrichment analysis

To assess the function of DEGs in the ceRNA network in tumorigenesis, we performed Gene Ontology (GO) annotation and Kyoto Encyclopedia of Genes and Genomes (KEGG) pathway analyses using the clusterProfiler package of R software [17]. *P*-value < 0.05 was set as the cut-off criterion.

## Results

### Identification of differentially expressed genes

**Figure 1:**
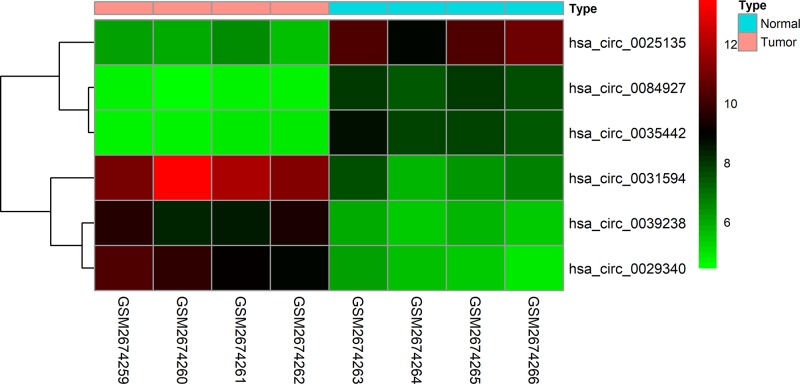
Heat map of the 8 differentially expressed circRNAs of the GSE100186 dataset

**Figure 2:**
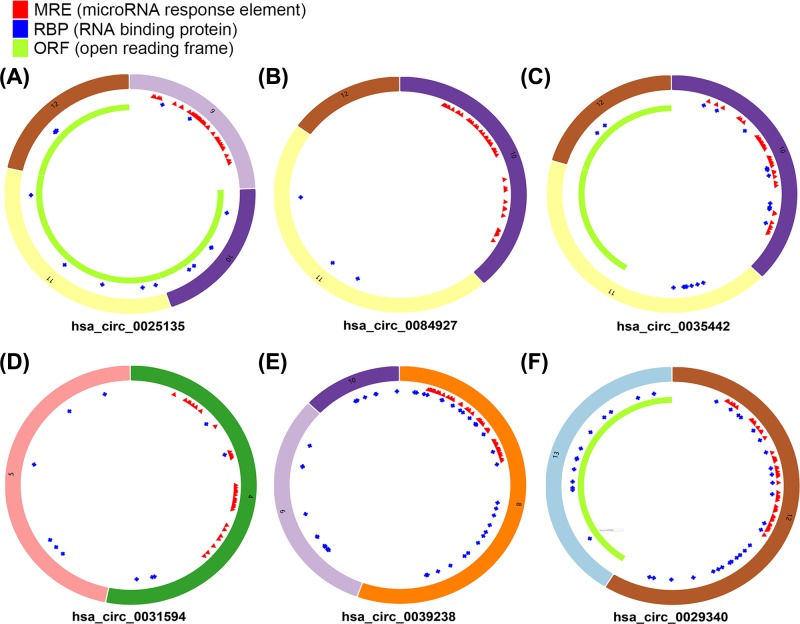
Structural patterns of the 6 circRNAs by the Cancer-Specific CircRNA (CSCD, http://gb.whu.edu.cn/CSCD/) (**A**) hsa_circ_0025135, (**B**) hsa_circ_0084927, (**C**) hsa_circ_0035442, (**D**) hsa_circ_0031594, (**E**) hsa_circ_0039238, (**F**) hsa_circ_0029340.

**Figure 3:**
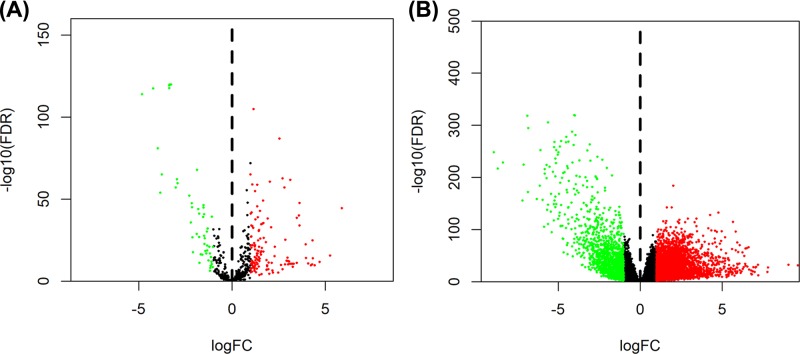
Volcano plot of differentially expressed miRNAs (**A**) and mRNAs (**B**)

A total of 6 differentially expressed circRNAs (DEcircRNAs) were screened from GSE100186 dataset, including 3 up-regulated and 3 down-regulated circRNAs (Figure 1). The basic characteristics of the 6 circRNAs are listed in Table 1. Their basic structural patterns are shown in Figure 2. The DEmiRNAs and DEmRNAs, obtained from the TCGA database, were analyzed across RCC tissues and adjacent normal tissues; we identified 187 DEmiRNAs (135 up-regulated and 52 down-regulated), and 5029 DEmRNAs (3681 up-regulated and 1348 down-regulated) (Figure 3A,B).

**Table 1:** Basic characteristics of the 6 differently expressed circRNAs

circRNA ID	Position	Genomic length	Strand	Best transcript	Gene symbol	Regulation
hsa_circ_0031594	chr14:34398281-34400421	2140	–	NM_022073	EGLN3	Up
hsa_circ_0029340	chr12:125292306-125294835	2529	–	NM_005505	SCARB1	Up
hsa_circ_0039238	chr16:47162235-47165936	3701	–	NM_018092	NETO2	Up
hsa_circ_0084927	chr8:95676924-95677424	500	+	NM_017697	ESRP1	Down
hsa_circ_0035442	chr15:58284902-58287337	2435	–	NM_001206897	ALDH1A2	Down
hsa_circ_0025135	chr12:6458115-6465046	6931	–	NM_001159576	SCNN1A	Down

### Construction of the ceRNA network

**Figure 4:**
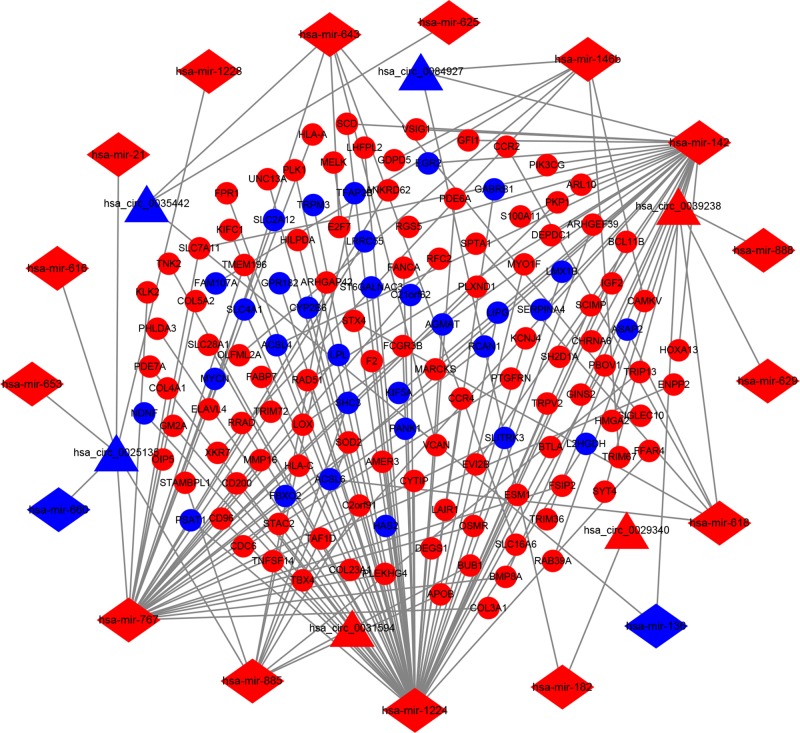
The ceRNA network of circRNA–miRNA–mRNA in RCC The network consists of 6 circRNA nodes, 17 miRNA nodes, and 134 mRNA. Triangles indicate circRNAs, diamonds indicate miRNA, and ellipses indicate mRNA. The nodes highlighted in red and blue represent up-regulation and down-regulation, respectively.

We used the 6 DEcircRNAs, retrieved from the CircInteractome database, and identified 90 circRNA–miRNA pairs. After intersecting with the DEmiRNAs, only 19 circRNA–miRNA pairs, including 6 circRNAs and 17 DEmiRNAs, remained. Furthermore, 833 mRNAs, predicted by both databases (miRTarBase and TargetScan), were identified; these were compared with the 5028 DEmRNAs and only overlapping genes were selected as candidate genes. Results indicated 134 DEmRNAs to be involved in ceRNA network. Finally, we constructed a ceRNA network based on 6 circRNA nodes, 17 miRNA nodes, and 134 mRNA nodes in RCC (Figure 4).

### Protein–protein network analysis

**Figure 5:**
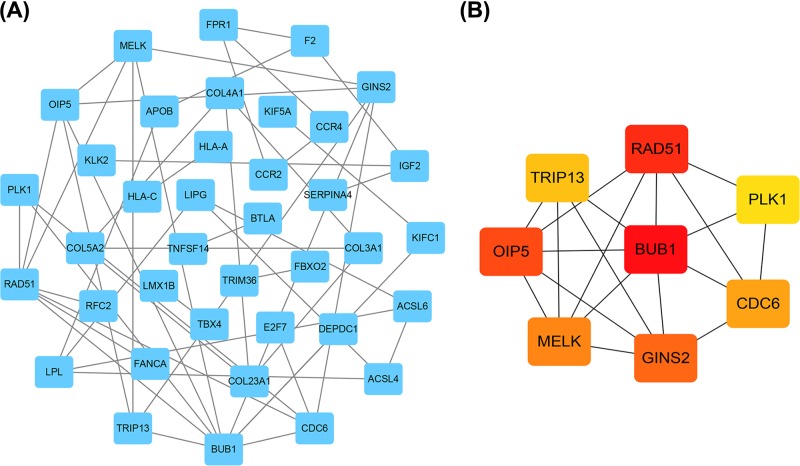
Identification of hub genes from the PPI network (**A**) PPI network of 134 genes, consisting of 37 nodes and 50 edges. (**B**) PPI network of 8 hub genes extracted from (A). The node color changes gradually from yellow to red in ascending order according to the log_2_(foldchange) of genes.

**Figure 6:**
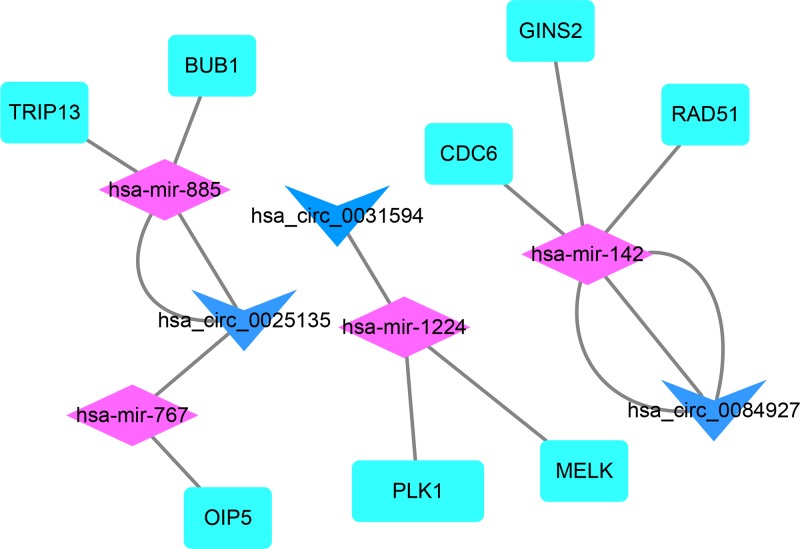
CircRNA–miRNA–hub gene network The network consists of 3 circRNAs, 4 miRNAs, and 8 hub genes. Vs indicate circRNAs, diamonds indicate miRNA, and round rectangles indicate mRNA.

Based on the DEmRNAs, PPI network was constructed, involving 37 nodes and 50 edges (Figure 5A). In order to identify hub genes in the process of RCC carcinogenesis, closeness centrality of DEmRNAs was calculated using cytoHubba plugin, and the top 8 hub genes were found to be BUB1, RAD51, GINS2, CDC6, MELK, OIP5, PLK1, and TRIP13 (Figure 5B). We next constructed a circRNA–miRNA–hub gene sub-network (Figure 6), including 8 ceRNA regulatory modules.

### Functional assessment of DEmRNAs

Our data revealed that the mRNAs associated with biological process (BP) included those in regulation of mitotic metaphase/anaphase transition, regulation of metaphase/anaphase transition of cell cycle, metaphase/anaphase transition of mitotic cell cycle, metaphase/anaphase transition of cell cycle, and regulation of mitotic sister chromatid separation. Meanwhile, the mRNAs related to cellular components (CC) were most relevant to chromosomal region (*P* <0.05). In terms of molecular function (MF), mRNAs were mostly enriched in catalytic activity, and acting on DNA (*P* <0.05). The top five GO terms are indicated in Table 2, according to the *P*-value. KEGG analysis showed cell cycle pathway to be associated with the mRNAs (*P* <0.05).

**Table 2:** The top 5 GO terms enriched by DEmRNA involved in the ceRNA network

Categories	Terms	Description	*P*-value	*P*-adjusted	Genes	Counts
BP	GO:0030071	Regulation of mitotic metaphase/anaphase transition	1.05E-06	7.64E-05	BUB1/CDC6/TRIP13	3
	GO:1902099	Regulation of metaphase/anaphase transition of cell cycle	1.11E-06	7.64E-05	BUB1/CDC6/TRIP13	3
	GO:0007091	Metaphase/anaphase transition of mitotic cell cycle	1.26E-06	7.64E-05	BUB1/CDC6/TRIP13	3
	GO:0044784	Metaphase/anaphase transition of cell cycle	1.34E-06	7.64E-05	BUB1/CDC6/TRIP13	3
	GO:0010965	Regulation of mitotic sister chromatid separation	1.50E-06	7.64E-05	BUB1/CDC6/TRIP13	3
CC	GO:0098687	Chromosomal region	0.000331	0.007594	BUB1/RAD51/OIP5	3
	GO:0005657	Replication fork	0.00037	0.007594	RAD51/GINS2	2
	GO:0000794	Condensed nuclear chromosome	0.000716	0.009784	BUB1/RAD51	2
	GO:0000775	Chromosome, centromeric region	0.002878	0.024971	BUB1/OIP5	2
	GO:0000793	Condensed chromosome	0.003585	0.024971	BUB1/RAD51	2
MF	GO:0140097	Catalytic activity, acting on DNA	0.003358	0.032713	RAD51/GINS2	2
	GO:0043138	3′-5′ DNA helicase activity	0.005912	0.032713	GINS2	1
	GO:0043142	Single-stranded DNA-dependent ATPase activity	0.005912	0.032713	RAD51	1
	GO:0000400	Four-way junction DNA binding	0.007272	0.032713	RAD51	1
	GO:0034185	Apolipoprotein binding	0.007272	0.032713	LPL	1

## Discussion

Abnormal expression of non-coding RNAs plays an important role in the development and progression of multiple tumors [18,19]. With the advent of high-throughput sequencing technologies in recent years, along with the continuous maturity and development of bioinformatics, circRNAs have been increasingly identified in multiple species. The discovery of circRNA expands our knowledge of the types and functions of non-coding RNA family. Several studies have unveiled the mechanism of participation of circRNAs in the regulation of malignant biological processes [20,21]. CircRNAs have been shown to be associated with the development of different tumors, thus implying their potential to serve as biomarkers of malignancies [22–24]. However, the exact role of circRNAs in RCC still remains largely unclear. In the present study, we first integrated circRNA, miRNA, and mRNA data of RCC tissues and non-tumor tissues from Gene Expression Omnibus (GEO) datasets and TCGA database, and constructed the circRNA–miRNA–mRNA regulatory network.

Several studies have shown the expression level of circRNA to be dysregulated in RCC, and to be associated with pathogenesis and prognosis, thus suggesting them as potential tumor-associated biomarkers [25–27]. Wang et al. [25] analyzed 52 pairs of RCC samples and normal renal samples and found hsa_circ_0001451 to be significantly down-regulated in RCC tissues, besides being linked to clinicopathological features and OS. *In vitro* silencing of Hsa_circ_0001451 promoted tumor growth; therefore, hsa_circ_0001451 may be a potential prognostic biomarker for RCC. Similarly, elevated circPCNXL2 was observed in RCC tissues and was associated with poor OS of patients with RCC. Knockdown of circPCNXL2 inhibited proliferation and *in vitro* invasion, and reduced tumor growth *in vivo*. Furthermore, circPCNXL2 was found to bind to miR-153 as an miRNA sponge to regulate the expression of ZEB2 in RCC progression [26]. In our study, a total of 6 circRNAs (hsa_circ_0084927, hsa_circ_0029340, hsa_circ_0035442, hsa_circ_0025135, hsa_circ_0039238, and hsa_circ_0031594) were identified to be involved in the ceRNA network. Among them, hsa_circ_0084927 was found to be up-regulated in lung adenocarcinoma-associated malignant pleural effusion and was used to construct the ceRNA network [28]. However, none of the other 5 circRNAs has yet been reported.

MiRNAs regulate approximately 60% of human genes, including proto-oncogenes and tumor suppressors, suggesting the correlation between miRNAs and tumors. MiRNAs form a large class of endogenous non-coding RNAs that are 19–25 nucleotides in length, regulating cell proliferation, differentiation, apoptosis, and migration [29]. They can bind to the 3′ untranslated region (UTR) of the target gene by means of partial pairing of base pairs to inhibit the expression of target gene at post-transcriptional level [30]. In the present study, we identified 17 DEmiRNAs in the ceRNA network. Among them, several miRNAs have been reported to play important roles in the initiation and development of RCC, such as miR-182, miR-136, and miR-629 [31–33]. To further identify the key circRNAs participating in the regulatory network, we established the PPI network, thereby screening 8 hub genes. GO and KEGG pathway analysis suggested these DEmRNAs to be involved in many important tumor-associated biological functions and pathways. Furthermore, we established circRNA–miRNA–hub gene network, including 8 circRNA–miRNA–mRNA axes. However, since the results are based on bioinformatics, further in-depth studies would be recommended to verify the possible role of the 8 axes in RCC.

## Conclusions

We obtained the expression profiles of RNAs and constructed ceRNA networks by analyzing the data from GEO and TCGA. Our study provides novel insights into the circRNA-related ceRNA network in RCC and suggests potential therapeutic targets.

## Abbreviations

CircRNA: circular RNA
RCC: renal cell carcinoma
